# Process Optimization and Efficacy Assessment of Standardized PRP for Tendinopathies in Sports Medicine: Retrospective Study of Clinical Files and GMP Manufacturing Records in a Swiss University Hospital

**DOI:** 10.3390/bioengineering10040409

**Published:** 2023-03-25

**Authors:** Patrick Sebbagh, Nathalie Hirt-Burri, Corinne Scaletta, Philippe Abdel-Sayed, Wassim Raffoul, Vincent Gremeaux, Alexis Laurent, Lee Ann Applegate, Gerald Gremion

**Affiliations:** 1 Regenerative Therapy Unit, Plastic, Reconstructive & Hand Surgery Service, Lausanne University Hospital, University of Lausanne, CH-1066 Epalinges, Switzerland; patrick.sebbagh@gmail.com (P.S.); nathalie.burri@chuv.ch (N.H.-B.); corinne.scaletta@chuv.ch (C.S.); philippe.abdel-sayed@chuv.ch (P.A.-S.); wassim.raffoul@chuv.ch (W.R.); alexis.laurent@lambiotechnologies.com (A.L.); gerald.gremion@bluewin.ch (G.G.); 2 Lausanne Burn Center, Lausanne University Hospital, University of Lausanne, CH-1011 Lausanne, Switzerland; 3 DLL Bioengineering, STI School of Engineering, Ecole Polytechnique Fédérale de Lausanne, CH-1015 Lausanne, Switzerland; 4 Sport Medicine Unit, Division of Physical Medicine and Rehabilitation, Swiss Olympic Medical Center, Lausanne University Hospital, University of Lausanne, CH-1011 Lausanne, Switzerland; vincent.gremeaux@chuv.ch; 5 Manufacturing Department, LAM Biotechnologies SA, CH-1066 Epalinges, Switzerland; 6 Center for Applied Biotechnology and Molecular Medicine, University of Zurich, CH-8057 Zurich, Switzerland

**Keywords:** cell therapies, good manufacturing practices, musculoskeletal regenerative medicine, optimized manufacturing, orthobiologics, platelet-rich plasma, process standardization, sports medicine, tendinopathies, transfusion medicine

## Abstract

Platelet-rich plasma (PRP) preparations have recently become widely available in sports medicine, facilitating their use in regenerative therapy for ligament and tendon affections. Quality-oriented regulatory constraints for PRP manufacturing and available clinical experiences have underlined the critical importance of process-based standardization, a pre-requisite for sound and homogeneous clinical efficacy evaluation. This retrospective study (2013–2020) considered the standardized GMP manufacturing and sports medicine-related clinical use of autologous PRP for tendinopathies at the Lausanne University Hospital (Lausanne, Switzerland). This study included 48 patients (18–86 years of age, with a mean age of 43.4 years, and various physical activity levels), and the related PRP manufacturing records indicated a platelet concentration factor most frequently in the range of 2.0–2.5. The clinical follow-up showed that 61% of the patients reported favorable efficacy outcomes (full return to activity, with pain disappearance) following a single ultrasound-guided autologous PRP injection, whereas 36% of the patients required two PRP injections. No significant relationship was found between platelet concentration factor values in PRP preparations and clinical efficacy endpoints of the intervention. The results were in line with published reports on tendinopathy management in sports medicine, wherein the efficacy of low-concentration orthobiologic interventions appears to be unrelated to sport activity levels or to patient age and gender. Overall, this study confirmed the effectiveness of standardized autologous PRP preparations for tendinopathies in sports medicine. The results were discussed in light of the critical importance of protocol standardization for both PRP manufacturing and clinical administration to reduce biological material variability (platelet concentrations) and to enhance the robustness of clinical interventions (comparability of efficacy/patient improvement).

## 1. Introduction

Tendinopathies are debilitating and painful affections, which often have severe consequences on the daily activities (e.g., temporary or permanent interruption of work) of patients and on healthcare systems [1,2,3,4]. Frequently developed following overuse/overload injuries (i.e., professional or sports-related), tendinopathies mostly affect the rotator cuff, Achilles tendon, tibialis posterior, common wrist extensor, and patellar tendons [1,3,5]. Within the aging populations, sports-related (i.e., recreational or elite levels) tendon overuse drastically increases the risks and prevalence of injury [1,2]. Due to tissular hypocellularity and hypovascularity, the intrinsic and physiological healing ability of tendons is slow and inefficient [1]. This process becomes even slower with increasing patient age and can be disturbed by physical activity, especially in cases when sports-related practices exceed tissue recovery abilities. Lengthy recuperation results in direct (i.e., healthcare costs) and indirect (e.g., productivity loss) financial burdens on society [3,4]. Chronic tendon lesions are often degenerative in nature, yet they do not necessarily implicate inflammatory components [2,5].

If the tendon tissue’s extracellular matrix (ECM) structure is damaged, physiological repair processes may result in the synthesis of disorganized matrix, potentially leading to fibrin strand breakage (e.g., fibrillar slippage, breakage of cross-linking, and fibrillary rupture) [5]. The resulting structural anomalies may lead to a reduction in the axial strength and elasticity of the affected tissue, thus favoring the recurrence of lesions. Standard therapeutic management of sub-critical tendon defects rely on well-managed analgesia (e.g., prescription of NSAIDs) and physical therapy (e.g., eccentric muscle work) [3,4,5]. In case of important structural damage and low probability of spontaneous healing, invasive therapeutic interventions may be considered (e.g., tendon suture, autografts, allografts, and synthetic prostheses) [1,5]. As an intermediate approach, injectable hyaluronan-based hydrogels have demonstrated multiple therapeutic benefits in tendon-related affections [6]. Furthermore, diverse regenerative medicine approaches have recently been investigated to improve tendinopathy management, including stem cell therapies, tissue graft bioengineering, and the use of orthobiologics [7,8,9,10,11,12,13,14]. Notably, recent regulatory shifts and quality-oriented constraints related to cytotherapies have prompted the deployment of important efforts toward the development of cell-derived and cell-free therapeutic management options for tendinopathies [15,16,17,18,19,20,21,22]. Parallelly, the medical use of autologous platelet-rich plasma (PRP) preparations in orthopedics and in sports medicine for tendon and ligament affections has drastically increased over the past two decades [23,24,25,26,27].

Autologous PRP therapy for tendon affections requires a local ultrasound-guided injection of a processed blood extract, which has been obtained by minimal manipulation (e.g., differential centrifugation) from patients’ blood. Such extracts are composed of concentrated platelets in autologous plasma following the removal of erythrocytes and lymphocytes [28,29,30]. Standard native blood sample volumes to be drawn from patients for PRP therapies in sports medicine range from 20 mL to 40 mL, with average blood platelet counts of 200,000 ± 75,000 platelets/µL. Following processing, the plasma, inflammatory agents, platelets, and plasma proteins are conserved in the PRP preparation [25,28,31]. Delivery of supra-physiological concentrations of growth factors and cytokines found in PRP (i.e., following platelet breakdown) often stimulates an accelerated and physiological regenerative response at the site of the tissular injury. Currently, PRP is commonly used in surgery and regenerative medicine to improve the recovery potential of soft tissues (e.g., ligaments, tendons, cartilage, and nerves) and bones [32,33,34,35,36,37]. Its clinical use has also been extensively reported for cutaneous wounds, burns, and skin donor sites, as well as for esthetic applications (e.g., cutaneous and capillary) [28,38,39,40,41,42,43]. Finally, PRP applications in sports medicine have been shown to enhance recovery following tendon lesions and, in particular, result in shortened overall recovery periods for athletes [7,8,33].

Importantly, highly contradictory reports and analyses of PRP clinical efficacy are available in current scientific literature sources, when considering all therapeutic indications and manufacturing methods [12,14,24,25,27,38,40,44,45]. The critical importance of standardizing sample processing workflows and therapeutic protocols has been identified in order to obtain maximal benefits from orthobiologic treatments. However, a vast array of PRP production protocols is available on the market, which can potentially explain several aspects of the observed variability in clinical efficacy outcomes [44,46,47]. Previous literature reviews have shown that actual PRP platelet concentration factors vary from 2 to 12 times in value, wherein extreme values are reported to be as low as 0.52 times the baseline ratio of platelets to whole blood [23,24,44]. Such diversity in PRP product attributes may be linked to the specific manufacturing systems used (e.g., commercial kits), the volumes of drawn blood, the starting cellular concentrations, the anti-coagulation techniques, and patient-related factors (e.g., comorbidities, age, circulatory and nutritional status, and drug use) [44]. While PRP manufacturing standardization has become a main clinical-oriented focus point, regulatory scrutiny is directed at the PRP manufacturing systems, rather than at the exact manufacturing process or the final PRP product attributes [48,49,50,51,52,53,54,55]. Importantly, it should be noted that higher platelet concentrations in the final PRP preparation do not systematically correlate with enhanced clinical efficacy as it seems that saturation can occur, and the timing of administration may also have an impact on overall tissue healing parameters [24,44]. Finally, there is no widespread consensus regarding post-injection management for PRP in sports medicine, wherein rehabilitation plays a crucial role [56].

This retrospective study (i.e., covering the 2013–2020 period) investigated the standardized GMP manufacturing and sports medicine-related clinical use of autologous PRP for tendinopathies at the Lausanne University Hospital (CHUV, Lausanne, Switzerland). The primary objective of the study was to evaluate PRP injection efficacy in patients treated at the Sports Medicine Unit for tendon-related affections based on the clinical files and GMP manufacturing records of PRP preparations. As previously reported, the in-house GMP manufacturing of PRP preparations at CHUV has enabled rapid clinical management of tendinopathies, cutaneous burns, and arthrosis lesions in multiple institutional departments [31]. From a technical point-of-view, the developed and standardized PRP manufacturing protocol increases the platelet concentration by a factor of 2–3 times from 20 mL of native blood, using a two-step centrifugation method within a GMP platform [31]. A secondary objective of this study was to determine if a relationship could be identified between individual PRP concentration factors and patient-reported efficacy of the therapeutic intervention. Therefore, individual patient follow-up data were analyzed for the determination of the number of received PRP injections and for the assessment of patient clinical conditions at two, three, four, and five weeks post-PRP administration. Overall, the presented information outlines the key steps of PRP protocol standardization (i.e., manufacturing process and clinical administration), which were designed to reduce variability in final platelet concentrations and to enhance the quality of clinical interventions.

## 2. Materials and Methods

### 2.1. Study Design and Ethics Committee Approval of the Retrospective Study

The present retrospective study was reviewed and approved by the Vaud State Ethics Committee (i.e., authorization N°CER-VD-ID#2022-00305, 2022) and was conducted following the principles of the Declaration of Helsinki and applicable Swiss laws [57]. In this study, patient data from clinical cases of tendinopathy were used to assess the effectiveness of autologous PRP injections. The primary objective of the study was to determine the level of efficacy (i.e., categorized as “positive” or “non-positive” evolution) of PRP injections for the therapeutic management of tendinopathies. The secondary objective of the study was to determine if there was a relationship between the concentration factor of platelets in the administered PRP preparations and patient-reported efficacy of the intervention, which was assessed based on the patients’ clinical status at the time of the 2-, 3-, 4-, and 5-week follow-up contacts after the PRP injection. Specifically, the data were analyzed for the following purposes:

(i) Discriminate the success rate according to the age of the patients at the time of the PRP treatment.
(ii) Highlight whether PRP could help in resuming physical activity faster in younger versus older patients.
(iii) Determine how many PRP injections were necessary on average.
(iv) Highlight the nature and volume of the resumed physical activity in the studied patient population.

In order to obtain appropriate data and information within the scope of this retrospective study, the specific elements of the request submitted to the local ethics committee comprised the following:

(i) Number of PRP applications required for healing.
(ii) Ratio of platelet concentration in the PRP injection to platelet concentration in native blood (i.e., platelet concentration factor) for each patient.
(iii) Possible relationship between the number of PRP injections required and the platelet concentration factor.
(iv) Potential treatment-related adverse events as detected.
(v) Patient clinical evolution following the PRP treatment.

Given the exploratory nature of this retrospective study, the sample size calculation was not based on formal statistical methods. The sample size was evaluated according to an internal feasibility study, which showed that 75 patients with diagnosed tendinopathies were treated with autologous PRP injections since the implementation of the procedure in 2013 at the CHUV and would be eligible for the retrospective study.

### 2.2. Clinical Data Gathering and Processing

Patient clinical files were investigated at a preliminary level for cases of tendinopathy treatment by autologous PRP injection at the Sports Medicine Unit, since the implementation of the procedure at the CHUV in 2013 and until December 2020. The inclusion criteria restricted the search to the adult patient population (i.e., ≥18 years of age) that received a PRP injection treatment for tendinopathy. The exclusion criteria disqualified pediatric patients (i.e., <18 years of age) and patients who refused to be included in any research or have their data anonymously analyzed. Overall, 75 patients were initially included in the study. Further investigations led to the exclusion of several cases based on the absence or refusal of consent provision or on the presence of documented epicondylalgia. Finally, a total of 48 patients were retained for an in-depth analysis within this retrospective study.

Demographic, radiological, biological, and clinical data were collected and analyzed from computerized patient records (i.e., Soarian, Archimedes, PACS). The patient data notably included clinical and anamnestic histories, clinical and health observations, ultrasound image reports, choice of treatment, application of treatment, and ambulatory follow-up. In order to investigate the potential relationship between platelet concentration factors and PRP treatment efficacy, the patients were classified according to the qualitative assessment of their clinical state and the ultrasound appearance of their tendon tissular lesion.

### 2.3. GMP Manufacturing Process for Autologous PRP at the CHUV

All patients included in the present retrospective study received final autologous PRP products prepared by an in-house GMP platform, following a standardized CHUV-UTR protocol [31]. The in-house GMP facilities at the CHUV were accredited by Swissmedic (i.e., Swiss national therapeutic products regulator) for the production and storage of biologicals, including PRP. The specific manufacturing method comprised aseptic open-container manipulation, a two-step serial centrifugation workflow, and conditioning of the final PRP products in patient-specific syringes. The step-by-step instructions for PRP manufacture following the CHUV-UTR protocol are further provided in the following section. The final PRP products were typically characterized by a 2–3-fold platelet concentration factor (i.e., compared to native blood) using a low starting blood volume (i.e., approximatively 20 mL). The CHUV-UTR protocol for GMP manufacturing of PRP was validated and qualified as simple, inexpensive, and rapid (i.e., short production time) [28]. Furthermore, the protocol offered reproducibility of quality and limited risks for patients, with low volumes of blood draw, and was, thus, considered an asset for autologous biological treatments at the CHUV.

### 2.4. Medical Method for Orthobiological Management of Tendinopathies in Sports Medicine at the CHUV

The use of autologous blood for PRP preparations significantly reduces the risk of cross-contamination compared to the use of third-party blood. This approach was, therefore, retained by the attending physicians at the Sports Medicine Unit in the CHUV. During patient anamnesis and diagnostic investigation, an ultrasound scan was performed on all the patients included in this study. The purpose of the ultrasound was to determine the precise site of the tissular injury and to qualify the type of tendon injury. Notably, several patients did not present any ultrasound-detectable lesions. Then, a native blood sample was drawn and was sent to the GMP production unit, along with a medical prescription for an autologous PRP preparation. Following the manufacture, the final PRP products were returned to the attending physicians within 2 h of blood collection.

The attending physicians excluded the presence of contra-indications for PRP treatment (i.e., presence of fever, infection, allergy, and other contra-indications) for each patient. For outpatient PRP local administration by injection, ultrasound guidance was chosen to ensure accurate positioning of the needle on the tendon tissue lesion. Ultrasound guidance (GE LOGIQ e, GE Medical Systems, Milwaukee, WI, USA) of the needle also ensured that sensitive tissues, such as nerves, vessels, and healthy tendons, were avoided. For the few patients who did not present any ultrasound-detectable tendon lesions, the available clinical information was used to determine the PRP injection site. For the administration of the PRP injection, the patients lied on an examination table in the dorsal, lateral, or ventral position depending on the area to be treated. Particularly, the most comfortable position for the patients was sought in order to avoid any movement during the procedure. The injection area was then locally disinfected using topical chlorhexidine, and the PRP preparation was injected. Standard antalgics were prescribed, and follow-up visits were specified as appropriate.

### 2.5. Statistical Assessment of Data

Quantitative variables that followed a Gaussian/normal distribution were analyzed and visualized using means and standard deviations. Categorical (i.e., qualitative) variables were represented by percentages and frequencies. The statistical calculations and/or data presentation were performed using Microsoft Excel and Microsoft PowerPoint (Microsoft Corporation, Redmond, WA, USA).

## 3. Results

### 3.1. Retrospective Study Workflow and PRP GMP Manufacture

**Figure 1:**
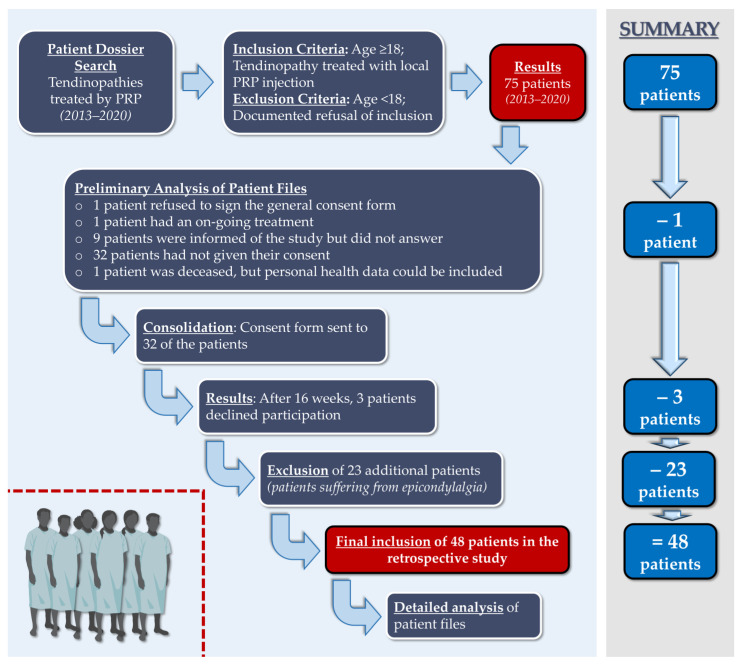
Overview of the retrospective study protocol (i.e., study workflow) for patient selection and inclusion for analysis. Data procurement was based on a retrospective review of patient dossiers for clinical cases of tendinopathies treated with local autologous PRP injections between 2013 and 2020 at the Sports Medicine Unit of the Lausanne University Hospital. A summary of the number of included patients during the selection procedure is presented in the column on the far right. PRP, platelet-rich plasma.

The methodological overview of the present retrospective study is presented in Figure 1, with a description of the patient inclusion/exclusion criteria and the processing of clinical files within the study workflow.

**Figure 2:**
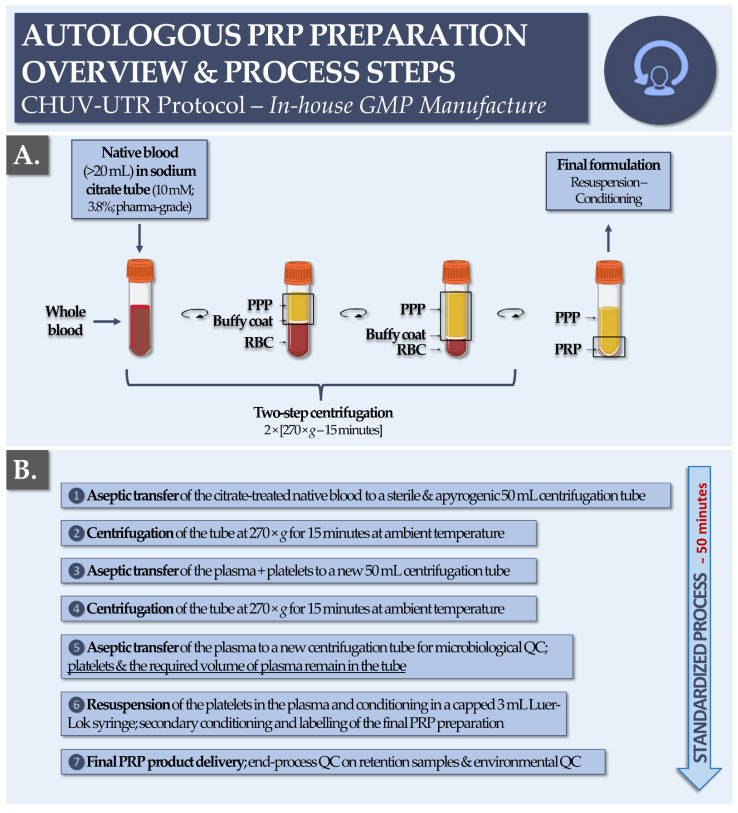
Illustrated and step-by-step process description for standardized autologous PRP manufacture under GMP at the Lausanne University Hospital. (**A**) Illustrated overview of autologous blood product processing, with two-step serial centrifugation for eventual platelet concentration. (**B**) Step-by-step description of the autologous blood product processing method for the obtention of the prescribed volume in the final autologous PRP product. Platelet counts were performed using the native blood sample and the final PRP product. CHUV, centre hospitalier universitaire vaudois; GMP, good manufacturing practices; PPP, platelet-poor plasma; PRP, platelet-rich plasma; QC, quality control; RBC, red blood cell; UTR, regenerative therapy unit.

For all patients included in this retrospective study (i.e., 48 patients, Figure 1), a complementary analysis of available PRP manufacturing records was performed. The exact method of autologous PRP manufacture by the in-house GMP platform is presented in Figure 2, with an illustrated and step-by-step process description.

### 3.2. Patient-Related Parameters and PRP Clinical Administration for Tendinopathies

**Figure 3:**
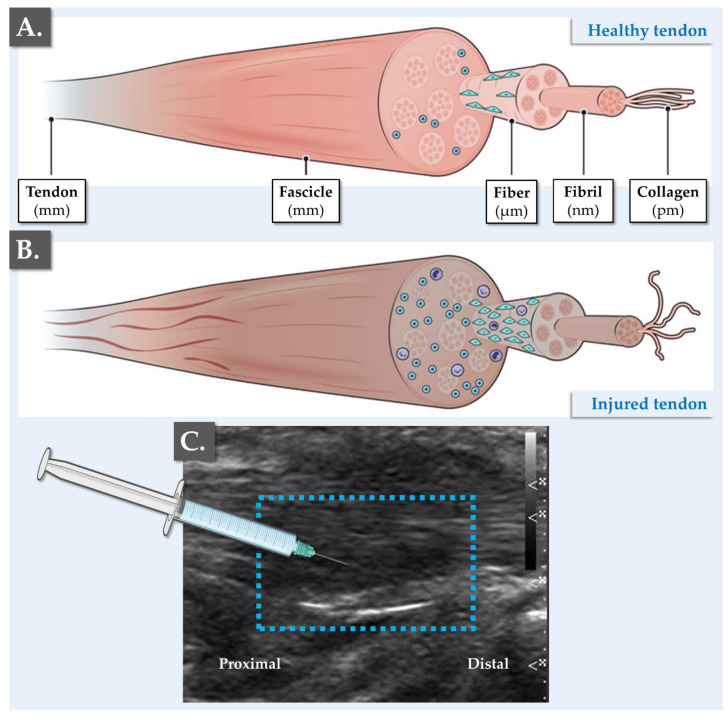
(**A**) Healthy tendon structure and components, including size scales. In normal conditions, the tendon ECM undergoes constant regeneration and re-modeling. (**B**) Unhealthy tendon structure and components. In case of vascular, inflammatory, degenerative pathology, or mechanical overload, tendon homeostasis is disrupted. This leads to a gradual accumulation of ECM damage and disorganization, with alteration of collagen architecture, glycosaminoglycan deposition, lipid accumulation, and heterotopic ossification. Compared to healthy tendons, diseased tendons have elevated collagen disorganization, smaller fibers, hypercellularity, increased cell rounding, elevated presence of immune cells, and increased denatured collagen. (**C**) Fissured Achilles tendon structure as observed by ultrasound, with illustration of the autologous PRP injection point. ECM, extracellular matrix; PRP, platelet-rich plasma.

The methodological aspects of the ultrasound-guided PRP administration for therapeutic tendinopathy management are presented in Figure 3, with anatomical description of the injured tendons and ultrasound records for a managed case of Achilles tendon fissure.

The demographic characteristics of the patients included in the present retrospective study are presented in Table 1.

**Figure 4:**
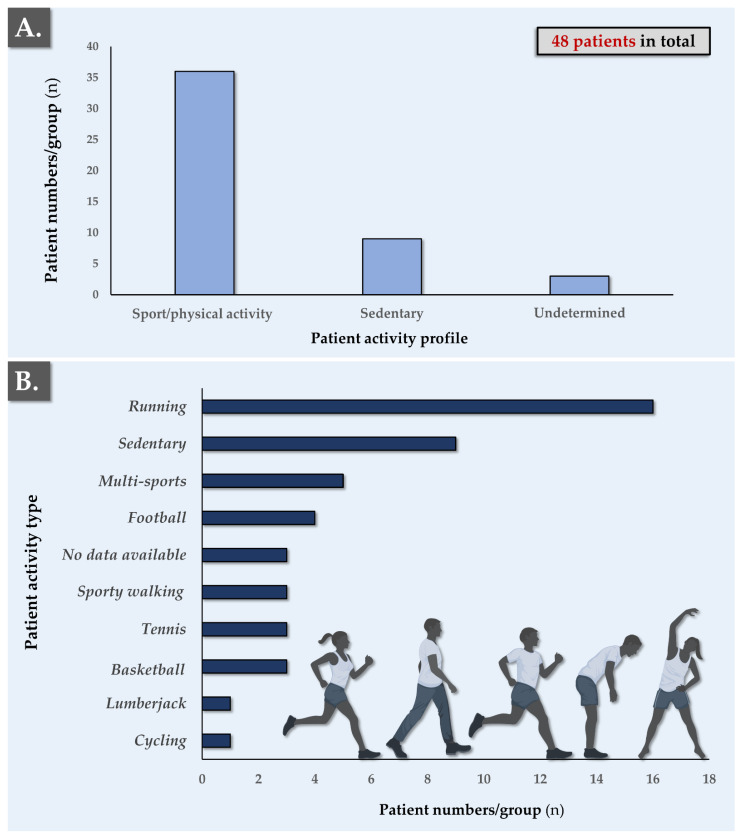
Activity-related parameters for the patients included in this retrospective study. (**A**) Patient physical activity status distribution at the time of autologous PRP treatment. (**B**) Type of sport or physical activity distribution for the included patients at the time of autologous PRP treatment. PRP, platelet-rich plasma.

Patient activity-related parameters were compiled and used to classify the patient sample into various groups, as presented in Figure 4.

An analysis of the clinical files revealed that a majority of the treated patients reported having a regular sport activity or an active physical professional activity (e.g., lumberjack), with 75% of the patients leading active lives (Figure 4). An examination of the background of the individual patients revealed that those who benefited from therapeutic autologous PRP injections were either highly involved athletes or patients who had previously remained refractory to several therapeutic approaches.

**Figure 5:**
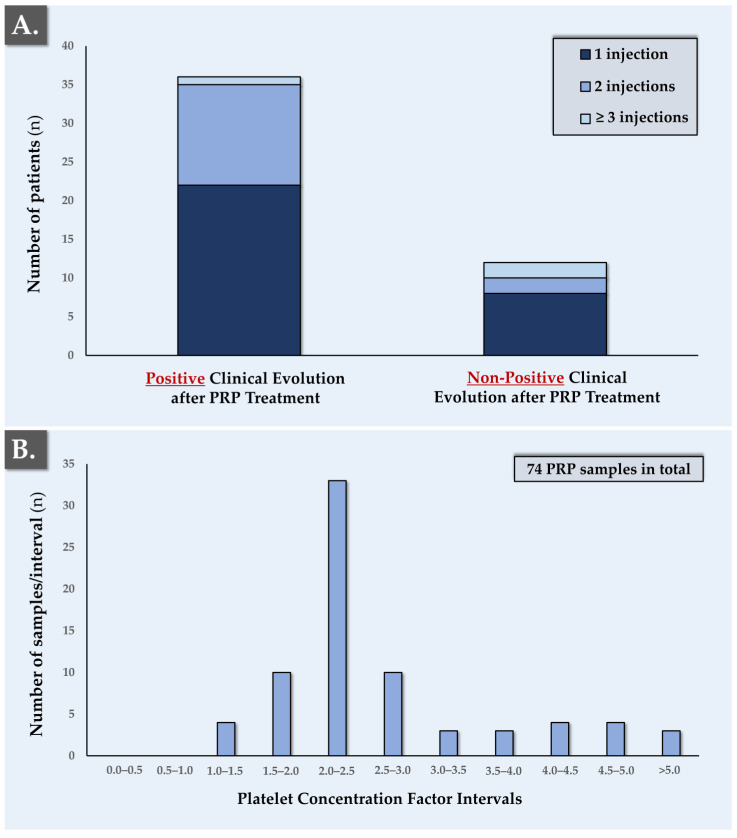
(**A**) Number of local autologous PRP injections received by the patients reporting positive or non-positive evolution of tendinopathy. It should be noted that, in both groups, some patients required more than three PRP injections. (**B**) Distribution of the platelet concentration factors in autologous PRP among the 48 patients, who received 74 PRP injections in total. PRP, platelet-rich plasma.

The diagnosis-related information gathered from the clinical files confirmed that all 48 patients presented severe tendon tissular alterations or a functional disorder. Regarding the follow-up of the patients after their PRP treatment and the evaluation of intervention efficacy, a positive evolution was defined as a complete recovery of physical condition, with complete disappearance of pain. An analysis of the patient follow-up data revealed that 61% of the patients who reported a positive evolution received a single autologous PRP injection (Figure 5A).

A total of 36% of the patients who reported a positive evolution required a second autologous PRP injection to maintain this positive evolution, and 3% of the patients who reported a positive evolution required three or more PRP injections to fully recover (Figure 5A). Overall, 36 patients reported a positive evolution following local PRP treatment, and 12 patients reported a non-positive evolution (Figure 5A).

### 3.3. PRP GMP Manufacturing Data Analysis and Clinical Efficacy Evaluation

The analysis of PRP manufacturing records for the patients included in this retrospective study revealed that the average native blood samples drawn from the patients for autologous PRP preparation were characterized as having a volume of 20 mL. The platelet concentration factors of the final autologous PRP products for clinical use were 2.79 on average, whereas the most frequent platelet concentration factor interval was of 2.0–2.5 in value (Figure 5B, Table 2).

Overall, no significant relationship was found between the final PRP product attributes (e.g., platelet concentration factor values) and efficacy-related outcomes in the analyzed dataset. On average, the patients who reported a positive evolution were younger than those who reported a non-positive evolution (Table 2). However, due to the absence of a statistically significant (i.e., with *p* < 0.05) difference in mean patient age values between the two groups, it was concluded that patient age was not a predictive criterion for autologous PRP treatment efficacy in the studied patient population.

An analysis of patient gender in relation to autologous PRP treatment efficacy revealed that males reported a positive evolution more often than females (i.e., 69.4%, Table 3).

However, no gender-related patient distribution differences were found in the group of patients who reported a non-positive evolution (Table 3). Interestingly, the data revealed that the six males who did not benefit from a positive evolution received only one PRP injection compared to an average of 1.6 PRP injections in the group that reported a positive evolution (Table 3). In comparison, the females who did not report a positive evolution received 2 PRP injections on average compared to the average of 1.6 PRP injections in the positive evolution group (Table 3). It was not possible to determine whether the group of male patients who did not improve became discouraged more quickly or whether their attending physician discontinued the treatment too soon. For the female patients, it was found that patient care was more sustained (Table 3).

Further investigation of the clinical patient files was performed for the patient group who reported a non-positive evolution following local autologous PRP treatment of tendinopathy. Within this group, there were three elderly patients (i.e., age > 70 years) and two overweight patients (i.e., BMI value > 25). There were also two sports teachers, a sedentary 41-year-old patient, two athletes aged 21 and 32, a patient who was applying for disability insurance benefits, and a patient for whom no further information was available. It should be noted that five patients within the non-positive evolution group presented a significant discrepancy between anamnestic complaints and ultrasound findings.

### 3.4. Standardized PRP GMP Manufacturing Process Statistical Evaluation

When considering the distribution of platelet concentration factors for the 48 included patients, relatively low values were most frequent (i.e., 33 patients in the 2.0–2.5 concentration factor group, Figure 5B). Despite the presence of extreme values (e.g., platelet concentration factors > 5.0), the variability in the platelet concentration factor value distribution was assessed as being low overall. Therein, the recorded values of platelet concentration factor in the autologous PRP were found to be tightly normally distributed around a mean value of 2.79 ± 1.34 (Table 2). Specifically, it was determined that 79.7% of the individual concentration factor values fell within one standard deviation of the mean value. For reference, a theoretical Gaussian distribution would comprise 68.27% of the individual values within one standard deviation of the mean value (Table S1). This reduced variability in platelet concentration factors when compared to a Gaussian distribution may be considered positively from a manufacturing process standardization point-of-view, especially for inherently variable biological starting materials, such as native blood and its derivatives.

Furthermore, intra-patient variability concerning platelet concentration factors was also studied to assess the robustness of the PRP manufacturing process. Therefore, an analysis of platelet concentration factor variability for each patient having received ≥2 autologous PRP injections was performed (i.e., 19 patients included in the analysis, representing 45 PRP preparations). In detail, the mean platelet concentration factor was determined for every patient, where applicable. Then, a gap analysis was performed for each patient to study the difference between the individual values of platelet concentration factor and the mean value of platelet concentration factor for the specified patient (Table S2). The results of the gap analysis are presented in Table 4.

The relatively low average results of the gap analysis presented in Table 4 can also be considered positively from a manufacturing process standardization point-of-view. Such analyses demonstrate that within a standardized GMP process for PRP manufacture, high reproducibility and consistency in autologous final product attributes (e.g., platelet concentration) could be attained. This aspect is especially important for the assurance of consistency in the quality and critical attributes of the final therapeutic product administered to a specific patient.

## 4. Discussion

### 4.1. Applicable Legal Bases and Advantages of GMP Autologous PRP Manufacture

The clinical use of autologous PRP has expanded rapidly in recent years despite the absence of a clear consensus on product preparation and characterization methodologies [14,33,44,51]. In particular, the high diversity observed in the domain of autologous PRP preparation and use may be partly attributed to the heterogeneity of clinical users (e.g., hospitals, private clinics and medical offices, and esthetic institutes) and manufacturing materials (i.e., commercial PRP kits) [27,44]. This in turn may potentially explain the highly contrasted clinical reports on orthobiologic intervention efficacy assessment, as clinical results are highly dependent on the therapeutic indication and on the PRP preparation/administration method [24,25,26,27,30,40,44]. In addition to the fragmented and inhomogeneous technical aspects of current autologous PRP preparation and use, the application of regulatory requirements is specific to member states in the European Union (EU), and several gray zones exist with regard to responsibility attribution [29,44].

In the case of Switzerland, the applicable legal bases for PRP preparation and use are highly similar to European frameworks. Therein, PRP is considered as a “non-standard medicinal product”, meaning that good manufacturing practice (GMP) standards must be applied [58]. Specifically, Directive 2005/62/EC clearly states that Good Practice Guidelines (GPG) based on the principles of GMP shall be implemented and defines the technical standards, which are very similar to GMP [48,49,50,53]. Such concepts and approaches are confirmed by the Swiss legislator in the Therapeutic Products Act (TPA, SR812.21) [58]. Authorizations from the national therapeutic products authority, Swissmedic, are therefore required for the GMP manufacturing of PRP preparations for clinical use [31]. Several exceptions and derogations to the aforementioned authorization regimen are specified, but Swiss private practices, clinics, or hospitals that do not rely on GMP autologous PRP manufacture must implement an appropriate ad hoc quality system [48,49,50]. Importantly, they must operate under defined procedures for the following activities:

(i) Training and qualification of personnel.
(ii) Validation of premises, equipment, testing procedures, and computerized systems.
(iii) Traceability assurance (i.e., proper labeling of samples and materials).
(iv) Storage and distribution of final products.
(v) Performance of self-inspections/audits for any complaints, recalls, and notifications to hemovigilance, along with implementation of appropriate corrective and preventive actions.

The purpose of the above-mentioned legal and regulatory procedures is to protect patients and to ensure that appropriate responsibility is assumed by clinicians for the assurance of the safety, quality, and efficacy of the administered autologous PRP treatments [48,49,50]. Overall and despite the elevated fixed costs of GMP manufacturing of autologous PRP products, optimal demonstration of compliance with regulatory standards is possible (i.e., use of a risk-based and process-oriented quality system) [31]. Therein, the autologous PRP manufacturing protocol developed by the Lausanne University Hospital (i.e., standardized CHUV-UTR protocol) has been validated from a regulatory standpoint within the scope of the Swissmedic authorization for the in-house GMP platform [28,31]. Importantly, ad hoc workflows for autologous PRP processing and trained personnel are available for the execution of medical prescriptions.

The validation of the GMP production platform and the qualification of the equipment and personnel contribute to guarantee the reproducibility of the quality of autologous PRP products, contrasting with extemporaneous preparation in a physician’s office using a kit. Importantly, the use of sub-optimal preparation and administration protocols can potentially jeopardize the clinical efficacy of PRP intervention and incur elevated morbidity and healthcare costs. Based on the sensitive nature of blood products and on the invasiveness of the PRP administration route (i.e., local injection), maximal safety and quality of the final product are ensured by the use of an appropriate GMP production platform [31].

### 4.2. PRP Manufacturing Process Standardization for Enhanced Therapeutic Quality

The demographic data of the included patient sample in the present retrospective study show high diversity in patients affected by tendinopathies (Figure 4, Table 1). Therefore, the use of a standardized manufacturing process for autologous PRP preparation is considered as a prerequisite for sound evaluation of intervention clinical efficacy. Indeed, for diverse types of cytotherapies and orthobiologics, the manufacturing process itself is the product and the object of standardization [31,59,60]. Importantly, it was reported that for PRP, no clear relationship existed between increased platelet concentrations in the finished product and enhanced clinical outcomes [23,24,25,26,30]. Such reports were confirmed by the results of the present retrospective study, in which non-significant trends of higher concentration factors were noted in the patient group with a recorded positive evolution (Table 2). Importantly, the recorded platelet concentration factors were found to be in line with previously published reports on the use of autologous PRP (i.e., 2–3 concentration factors, Table 2) [27,44].

It is important to mention that general or specific patient-related factors (e.g., use of aspirin, anti-inflammatory drugs, estrogens, or hormone replacement therapy) can potentially impact platelet activity and negatively influence autologous PRP treatment efficacy. Furthermore, the technical specifications of the PRP manufacturing process (e.g., centrifugation speed and time) are known to affect platelet reactivity and platelet yields in the finished PRP preparation, which can also potentially affect the therapeutic efficacy of the intervention [23,24,25]. While patient-related variability and variable factors are difficult or impossible to modify, manufacturing process-related specifications may be optimized and validated in order to minimize the detrimental impacts on platelet yields and activity in the PRP therapeutic preparation. Specifically, development efforts should be directed toward the use of processing and formulation methods that enable the obtention of PRP products characterized by high platelet yields and purity, along with high levels of therapeutic activity [23,27,44].

From a manufacturing and control point-of-view, further autologous PRP product standardization could be performed, notably by normalizing the platelet concentration in the final PRP product; however, several factors argue against such steps. Firstly, the low dispersion of recorded platelet concentration factor values and the documented high process robustness in this retrospective study indicated that limited technical gains could be procured by such an endpoint normalization (Figure 5, Table 4). Secondly, the additional processing steps incurred by such a normalization would result in higher direct manufacturing costs, as the process would require adaptation to individual samples (e.g., use of larger volume syringes and conditioning materials). Thirdly, the risk level (i.e., microbial or particulate contamination) would be increased with a non-standardized final step of PRP manufacturing (e.g., sample-specific dilution in plasma for platelet concentration adjustment). Finally, as the relationship between platelet concentration in PRP and clinical efficacy is tendential at best, no significant clinical gains in terms of product efficacy may be considered or would justify a further effort to standardize the final product itself. For all of the aforementioned reasons and due to the specific nature of clinical treatment with autologous PRP, it may be concluded that optimal quality and efficiency may be attained with the appropriate use of an ad hoc and standardized GMP manufacturing workflow (Figure 2).

An alternative technical approach to PRP final product standardization would be individual growth factor or cytokine content-based standardization, which presents both advantages and disadvantages [36,44]. On the one hand, selection of a single quality control marker (i.e., or a small panel of markers) to be used for PRP product standardization is technically feasible, with the use of specific and sensitive analytical tools, and may be considered advantageous over the current controls performed using iterative platelet enumeration from an analytical viewpoint. However, inclusion of such analytical steps would incur additional manufacturing costs and may eventually not be accurate or robust due to inter-patient (i.e., natural variability) and intra-patient (i.e., depending on the timepoint of blood collection) variability. Therefore, despite analytical precision advantages, high starting material variability and the need for the demonstration of enhanced clinical efficacy have not favored the widespread use of growth factor/cytokine-based autologous PRP product standardization [44].

Overall, the presented results confirmed the interest of using a standardized PRP manufacturing workflow, especially for patient cohorts presenting high demographic variability. Parallelly, it may be concluded that detailed investigation into patient demographic and diagnostic parameters is of prime importance for the appropriate demonstration of PRP intervention efficacy. By extension, the performance of large-scale retrospective studies on the therapeutic uses of PRP, taking into account all relevant demographic factors, could potentially enable the identification of predictive factors of standardized PRP intervention efficacy. This would, in turn, enable researchers to better define the applicable or optimal clinical pathway and treatment regimen using autologous PRP, thereby facilitating enhanced clinical success and rationalization of overall healthcare resources.

### 4.3. High Interest in Orthobiologics for Tendinopathy Management in Aging Populations

The overall prevalence of osteoarthritis, tendinopathy, and ligament injuries generally increases with age. Notably, rotator cuff tendinitis, rhizarthrosis, and carpal tunnel increase in women during perimenopause, without any direct link to physical activity or trauma [1,2,3,5,61]. In younger patients, physical activity or trauma-related injuries (e.g., cruciate ligaments, knee internal or external lateral ligaments, and Achilles tendon) are much more common [1,2,3,5]. Natural musculoskeletal tissue lesions are accentuated/aggravated following menopause or andropause [3,61]. Several points may be considered to tentatively explain these pathophysiological variations in connection with the endocrine status of patients. Specifically, estrogens and androgens have been shown to (i.e., directly or indirectly) influence cartilage tissue health [62]. Indirectly, as neurohormones, androgens affect behavior, mood, mental status, or cognition, and they impact on the willingness to undertake or exert physical effort [61,62]. Decreases in physical activity directly impact the release of growth hormone (GH), which can also be triggered by food intake and physical, psychic, or caloric stress during a fast [63]. Therefore, a decrease in metabolism associated with reduced physical activity favors an increase in patients’ BMI, further reducing GH secretion and production of IGF-1, which regulates somatic growth and plays a central role in skeletal growth [61,63].

With regard to age-related variations in tendon tissue healing, the decline of the vascular bed can also contribute to the progression toward less efficient tissular repair, as well as to the emergence of clinically perceivable pain for minor lesions. Inflammation and vasculature in tendinopathy have been poorly studied as neo-vessels seem to be very different from the normal vasculature formed during tissue growth and are not fully functional [5,64]. Overall, there are three major mechanisms of tendon degeneration:

(i) Overuse of tendons implicating ECM degradation (e.g., improper training, muscle imbalance, and overload by repetition or acute excessive exercise) [2,3].
(ii) Formation of neo-vessels related to exogenous stresses and stimuli (e.g., pharmacological treatments, smoking and alcohol consumption, environmental factors, and diet) [5,64].
(iii) Tissue aging related to endogenous factors (e.g., gender, body weight, hormonal factors, genetics, prior injury, and co-morbidities) [5,61,63].

Many biological parameters and lifestyle choices are involved in chronic tendinopathies, leading to multifactorial etiologies and alterations. Among the possible alterations to normal tissular functions are perturbations of nerve function and vascularization, cell density and phenotypes changes, cell–cell interactions, cell–matrix interactions, cytokine balance, and overall tendon matrix alterations (Figure 3) [5,64]. Based on the high complexity of tendon-specific pathologies and healing process, an autologous biological-based therapeutic approach (e.g., PRP injections) appears to be an optimal management option [65,66]. PRP is easily available in standard clinical settings and contains a variety of bioactive factors, such as PDGF, TGF, VEGF, IGF, or EGF, which are known to be actively involved in tissue healing [23,67,68]. Therefore, the use of autologous PRP may provide numerous beneficial outcomes for regenerative therapies in diverse clinical affections, especially if the manufacturing and administration processes are standardized [14,24,29].

Within autologous PRP treatments, high patient-related innate variability exists for the quantity and quality of platelets. A variety of reasons related to overall patient health, diet, genetics, and environmental factors may play a role in the efficacy obtained following the administration of autologous PRP [69,70,71]. Published reports have indicated that appropriate final platelet concentration in therapeutic PRP products is indication-specific [44,70]. Namely, a platelet concentrating factor of 7–12 is necessary to demonstrate efficacy in capillary transplantation, whereas musculoskeletal affections seem to respond with lower platelet concentrations and frequent PRP administrations [23,24,25,26,70,72]. Such reports were notably confirmed by the data gathered in the present retrospective study, where platelet concentration factors were most frequently found in the 2.0–2.5 interval (Figure 5). Therefore, indication-specific studies using standardized autologous PRP preparations appear to be a good starting point for devising optimal PRP dosing and administration regimens, with the endpoint objective being to enhance clinical efficacy and efficiency.

Taking all the abovementioned patient-related factors into account, it would seem logical to obtain poorer results in elderly and overweight patients with little or no physical activity and low androgen levels [61,62]. Such trends were partly confirmed by the detailed demographic analysis of the patients who reported a non-positive evolution following the PRP treatment (Figure 5). In this context, it could be advisable to consider different types of clinical regimens for autologous PRP enriched in platelets according to the age or the endocrinological status of patients, or to consider a chronic treatment pathway. This type of approach is considered to bear more potential for long-term success, reduced costs, and reduced morbidity when compared to more invasive techniques (e.g., autologous plasmapheresis) or alternative biological-based therapies, which require substantial manipulation.

### 4.4. Study Significance and Limitations

The present retrospective study sets forth the use of a fully GMP-compliant and accredited manufacturing platform for the preparation of finished autologous PRP products for clinical use. Although such practices should be applied in most cases according to the applicable legal bases in Europe and in Switzerland, very few clinical centers currently apply this level of quality and traceability for the preparation of autologous PRP. This is due to the well-known high variability in the content and application of the legal bases for PRP, as best shown within the European patchwork of laws, rules, and directives [29,51,52].

The first main study limitation consisted in the small size of the included patient sample (i.e., 48 patients included for retrospective analysis). This was mainly due to the limited number of patients treated for tendinopathies by PRP in Lausanne when compared to larger centers (e.g., in France or in the USA). However, this aspect is considered to be counterbalanced by the availability of high-quality original data pertaining to patient treatment and follow-up, as well as GMP manufacturing records for all the administered autologous PRP preparations. Notably, it is difficult to directly compare the present study to other published reports mainly due to the autologous PRP manufacturing method (i.e., GMP manufacturing of the final product versus the use of commercial PRP kits). However, similar trends were observed in the analyzed original data, such as the use of relatively low platelet concentration factors (e.g., 2–3 in value) for tendinopathy management and the absence of significant differences in efficacy related to final platelet concentrations.

Secondly, an accuracy issue was identified within the described GMP manufacturing process of autologous PRP with regard to the determination of platelet counts in whole blood and in the finished PRP preparations. Specifically, the mean platelet counts determined in whole blood were 73 ± 29 G/L for the samples included in the study, and 192 ± 96 G/L for the corresponding autologous PRP preparations. These values were found to be low with regard to normal whole blood platelet counts in the general population (i.e., 150–450 G/L) [47,73,74,75,76]. It was concluded that the enumeration values recorded in the GMP manufacturing batch records did not accurately reflect the platelet counts in whole patient blood as the included patients were not clinically qualified as being thrombocytopenic. The ad hoc investigations into the origins of this systematic accuracy issue enabled us to exclude platelet counting mistakes as all sample analyses were performed on a Sysmex XN-9000 automated hematology instrument. Therefore, it is most probably the sample processing itself (i.e., use of multiple small blood harvest tubes, use of sodium citrate instead of EDTA, and prolonged time periods between blood collection and analysis) that systematically resulted in the obtention of low platelet counts in the samples. Despite the identified accuracy issues for platelet enumeration, the precision of the retained method was confirmed, notably in the context of the intra-patient variability investigation, as presented and discussed in this article.

Importantly, as the present study focused on the attributes of the final autologous PRP products and specifically on the platelet concentration factors characterizing the obtained PRP, the aforementioned systematic accuracy issues within platelet enumeration bear virtually no consequences (i.e., use of a relative value or platelet ratio, and not absolute values) and have not resulted in any kind of systematic clinical failures. However, this important element constitutes a prime real-world and experience-based example of the current need for additional standardization of manufacturing and control activities for PRP products [44,77,78,79,80]. Specifically, this could technically be performed by optimizing sample preparation workflows (i.e., primary containers, anticoagulant type, and analytical timelines) or by using and validating platelet enumeration methods, which are not only precise but also accurate in a variety of clinical settings.

## 5. Conclusions and Perspectives

This retrospective study considered the standardized GMP manufacturing and sports medicine-related clinical use of autologous PRP for tendinopathy management in 48 patients. Importantly, the results of this study have demonstrated the effectiveness of standardized autologous PRP preparations for tendinopathies in sports medicine. As shown in the presented results and related data interpretations, manufacturing process standardization and holistic integration of patient-related factors are necessary for an appropriate evaluation of autologous PRP administration efficacy. Notably, the available GMP manufacturing records have shown that relatively low platelet concentration factors (i.e., 2.0–2.5) are sufficient to obtain a positive evolution in most of the treated clinical cases. The statistical discussions of the available data enable us to outline the manufacturing process repeatability and robustness, which are critical factors to ensure the continuous provision of high-quality orthobiologic care to each patient. Furthermore, a detailed analysis of the demographic factors of the patients who reported a non-positive evolution following PRP treatment provides some insights into how to further perform clinical pathway optimization in complex cases. Overall, the presented results confirm the central importance of process and protocol standardization for autologous PRP manufacturing/control and clinical administration. These elements are considered as prerequisites for the reduction of inter-individual variability and for the sound assessment of therapeutic intervention efficacy. Finally, the analyses of process-based and patient-related elements in the present study may be useful in the establishment of enhanced clinical workflows for autologous PRP therapies, aiming to reduce tendinopathy-related morbidity and costs and ensure long-term patient remission. To further enhance the orthobiologic treatment’s clinical efficiency for tendinopathies in sports medicine, relevant insights may be gained from best practices in transfusion medicine to ensure safe and appropriate PRP clinical use.

## Figures and Tables

**Table 1:** Demographic characteristics of the patients treated with local autologous PRP injection for tendinopathies at the Sports Medicine Unit of the Lausanne University Hospital and included in this retrospective study. The patient age data follow a normal distribution. PRP, platelet-rich plasma.

Year	2013	2014	2015	2016	2017	2018	2019	2020	Total
Patients (n)	1	7	17	4	4	5	5	5	48
Age of patients (years)	Average of 43.4 ± 16.6 years								
Patient age distribution (n) ^1^	≤45 years old: 26 patients (54%) 46–65 years old: 16 patients (33%) >65 years old: 6 patients (13%)								

^1^ The results indicate that approximately half of the included clinical cases in this study are likely to be unaffected by menopause or andropause, while the remaining patients are likely to be already affected by steroidal decline.

**Table 2:** Manufacturing data for the autologous PRP products. For the patients in the non-positive evolution group, a non-significant trend of lower (i.e., comparison of mean quantitative values) platelet concentration factor values is evident when compared to the concentration factor values of the positive evolution group. The platelet concentration factor data were found to be normally distributed. PRP, platelet-rich plasma; SD, standard deviation.

Patient Follow-Up	Number of Patients	Patient Age (Mean ± SD)	Platelet Concentration Factors (Mean ± SD)
All patients	48	43.3 ± 16.6	2.79 ± 1.34
Positive evolution	36	41.7 ± 22.9	2.92 ± 1.46
Non-positive evolution	12	47.5 ± 28.5	2.28 ± 0.28

**Table 3:** Patient gender distribution in relation to local autologous PRP treatment efficacy evaluation for the 48 patients included in this retrospective study. PRP, platelet-rich plasma.

Patient Follow-Up	Male Patients (n) Percentage of Subgroup (%) Average Number of PRP Injections (n)	Female Patients (n) Percentage of Subgroup (%) Average Number of PRP Injections (n)
Positive evolution	25	11
	69.4%	30.6%
	1.6	1.6
Non-positive evolution	6	6
	50.0%	50.0%
	1.0	2.0

**Table 4:** Results of the intra-patient gap analysis performed between individual and mean platelet concentration factor values. A total of 45 autologous PRP preparations were included in the analysis, corresponding to 19 patients. The data were found to be normally distributed. PRP, platelet-rich plasma.

Parameters	Experimental Results
Average gap value	14.0% ± 6.3%
Average gap value in male patients	18.7%
Average gap value in female patients	9.8%

## Data Availability

The data presented in this study are available upon reasonable request made in writing to the corresponding author.

## Supplementary Materials

The following supporting information can be downloaded at https://www.mdpi.com/article/10.3390/bioengineering10040409/s1. Table S1: Theoretical model for statistical discussion of experimental data; Table S2: Detail of the gap analysis performed for the assessment of autologous PRP GMP manufacturing process robustness.

## Abbreviations

BMI	body mass index
CHUV	centre hospitalier universitaire vaudois
EC	European Commission
ECM	extracellular matrix
EDTA	ethylenediaminetetraacetic acid
EGF	epidermal growth factor
EU	European Union
GAG	glycosaminoglycan
GH	growth hormone
GMP	Good Manufacturing Practices
GPG	Good Practice Guidelines
HA	hyaluronic acid
IGF	insulin-like growth factor
MD	medical device
NSAID	non-steroidal anti-inflammatory drugs
PDGF	platelet-derived growth factor
PPP	platelet-poor plasma
PRP	platelet-rich plasma
QC	quality control
RBC	red blood cells
SD	standard deviation
TGF	transforming growth factor
TPA	Therapeutic Products Act
USA	United States of America
UTR	regenerative therapy unit
VEGF	vascular endothelial growth factor
