# Supplementary material for: Process Optimization and Efficacy Assessment of Standardized PRP for Tendinopathies in Sports Medicine: Retrospective Study of Clinical Files and GMP Manufacturing Records in a Swiss University Hospital
Source: Bioengineering (Basel). 2023 Mar 25;10(4):409. doi: 10.3390/bioengineering10040409 (PMC10135571; doi:10.3390/bioengineering10040409)
Supplement: Supplementary file 1 [file bioengineering-10-00409-s001.zip › Supplementary Materials.pdf]

Supplementary Materials

1. Supplementary Tables

Supporting information to the data presented in Figure 5B is presented in Table S1.

Table S1. Theoretical model for the statistical discussion of the data presented in Figure 5B (i.e., platelet concentration factor distribution). The presented model shows that the experimental data is more tightly distributed around the experimental mean value (i.e., for both of the considered standard deviation intervals) than in a theoretical Gaussian distribution. PRP, platelet-rich plasma; SD, standard deviation.

**Intervals around Mean Values**	**Theoretical Data Bracket in a Pure Gaussian Distribution** [1] (% of individual values within the specified interval around the mean value)	**Experimental Data Bracket in the Platelet Concentration Factor Distribution (Figure 5B)** (% of individual values within the specified interval around the mean value)
Mean value ± 1 SD	68.27%	79.70%
Mean value ± 2 SD	95.45%	95.90%

[1] Natural normal distribution.

Supporting information to the data presented in Table 4 is presented in Table S2.

Table S2. Detail of the gap analysis performed for the assessment of the PRP GMP manufacturing process robustness (i.e., intra-patient variability), with the example of patient N°1. For each patient who received ≥ 2 PRP injections, individual platelet concentration factor values were compared to the mean platelet concentration factor value of the specified patient. The gaps between the individual values and the mean values were quantified and expressed as percentages. GMP, good manufacturing practices; PRP, platelet-rich plasma.

**Preparations for Patient N°1**	**Platelet Concentration Factor Value** (n)	**Mean Platelet Concentration Factor Value** (n)	**Gap Between the Individual Values and the Mean Value** (%) [1]
PRP Preparation N°1	2.46	2.38	3.30%
PRP Preparation N°2	2.31		-3.30%

[1] The absolute values characterizing the gaps were used for statistical treatment and obtention of the results presented in Table 4.
